# Beyond survival: Advancing symptom science across the adolescent and young adult cancer continuum

**DOI:** 10.1016/j.apjon.2026.101014

**Published:** 2026-08-03

**Authors:** Robert Knoerl, Victoria Wytiaz

**Affiliations:** a Department of Health Behavior and Clinical Sciences, University of Michigan School of Nursing, Ann Arbor, MI, USA; b Division of Hematology/Oncology, Department of Internal Medicine, University of Michigan Medical School, Ann Arbor, MI, USA

More than 1.3 million people aged 15–39 years were diagnosed with cancer worldwide in 2022.^1^ In the United States, 5-year relative survival exceeds 80% for many cancers diagnosed during adolescence and young adulthood.^2^ In this Editorial, the term “survivors of adolescent and young adult (AYA) cancer” refers broadly to people diagnosed with cancer between 15 and 39 years of age, including those receiving active treatment and those who have completed treatment. These individuals continue to experience physical and psychosocial challenges that differ from those encountered by children and older adults with cancer. These challenges extend beyond differences in cancer biology and treatment to developmental milestones related to education, employment, financial independence, relationships, and family life.^3^

Treatment-related symptoms, including fatigue, chemotherapy-induced peripheral neuropathy, cognitive difficulties, sleep disturbance, and impaired physical functioning, often remain long after treatment has ended and when unaddressed, can disrupt daily functioning, psychosocial well-being, and long-term quality of life.^4^^,^^5^ Beyond their immediate impact, persistent symptoms may also contribute to long-term morbidity. For example, AYA cancer survivors are at increased risk for cardiovascular disease compared to their peers without a history of cancer.^6^^,^^7^ This stems from the knowledge that AYA cancer survivors also have an increased incidence of associated risk factors, such as hypertension, dyslipidemia, and current smoking.^8^^,^^9^ These risk factors are often influenced by cancer and treatment-related lifestyle changes such as reduced physical activity or poor nutrition due to somatic symptoms such as fatigue or nausea or psychosocial symptoms such as anxiety or depression.^10^^,^^11^ As such, attention and intervention for such symptoms in this population have the potential to shape long-term morbidity and mortality.

The consequences of persistent symptoms extend beyond physical health. Qualitative evidence demonstrates that cancer treatment-related symptoms can alter how AYAs perceive themselves after cancer treatment, with cancer survivors describing treatment-related symptoms as making them feel older than their peers and serving as a reminder of the possibility of cancer recurrence.^4^ Long-term survivors diagnosed during adolescence or young adulthood may experience a greater burden of anxiety and depression than survivors diagnosed later in adulthood.^12^ The importance of addressing psychological symptoms is further underscored by evidence of an elevated risk of suicide in this population.^13^

Despite the substantial impact of persistent symptoms on quality of life, relatively few symptom management interventions have been specifically designed and tested for AYA cancer survivors.^14^, ^15^, ^16^, ^17^ This disconnect between improving survival and persistent symptom burden demands a shift in priorities. Oncology nursing research, embedded in a larger oncological care model, should move beyond describing symptom prevalence toward developing, testing, and implementing supportive care interventions that are developmentally appropriate and responsive to the priorities of AYAs themselves.

Developing effective interventions first requires understanding how AYAs wish to participate in supportive care research. Historically, enrollment of AYAs in cancer treatment trials has remained low; identified barriers include limited awareness of available studies.^18^, ^19^, ^20^ Less is known about participation in supportive care research. Emerging evidence suggests that AYAs value supportive care trials that minimize disruption to their daily lives while addressing symptoms they perceive as meaningful. In a choice-based conjoint analysis, the perceived importance of the study topic and the type of intervention were among the strongest determinants of willingness to participate. Participants generally favored non-pharmacologic interventions, flexible scheduling, remote data collection, shorter study visits, and electronic survey completion.^21^ These findings reinforce that supportive care research should incorporate the preferences of AYAs, whose competing responsibilities often include school, employment, and family obligations.

Integrative oncology represents one promising avenue for addressing persistent treatment-related symptoms among AYAs. Defined as the evidence-informed use of mind-body therapies, lifestyle interventions, and natural products alongside conventional cancer care,^22^ integrative oncology aligns well with AYAs' preferences for non-pharmacologic symptom management. Recent American Society of Clinical Oncology (ASCO)–Society for Integrative Oncology (SIO) guidelines recommend several integrative approaches for managing common cancer-related symptoms.^23^, ^24^, ^25^ Although evidence specific to AYAs remains limited (i.e., ASCO-SIO guideline recommendations are based largely on adult cancer survivors), there are several early feasibility studies of remotely delivered integrative oncology interventions, including self-administered acupressure^26^ and tailored music-based relaxation.^27^, ^28^, ^29^ However, while integrative oncology interventions such as acupressure demonstrate efficacy and feasibility in adults,^30^ pilot trials among AYAs are uncovering potential issues with engagement. For example, in a pilot trial of self-administered acupressure for cancer-related fatigue, recruitment, retention, and intervention fidelity were strong, yet only 38% of participants adhered to the prescribed intervention dose.^26^ Findings highlight that remote delivery alone may be unlikely to overcome the competing demands of adolescence and young adulthood. Future research should therefore focus not only on intervention efficacy, but also on optimizing intervention dose, behavioral support, and implementation strategies that promote long-term engagement.

A practical solution to both introducing AYA cancer survivors to symptom-focused research endeavors and engaging them throughout the process is in reimagining the AYA survivorship care model. Survivors of AYA cancer have reported unmet needs and a limited sense of belonging within conventional survivorship services.^31^ One approach to addressing these gaps is to strengthen AYA-focused survivorship care. Dedicated AYA survivorship clinics are one model, but care may also be delivered through embedded AYA services, nurse-led navigation, oncology–primary care co-management, or virtual survivorship programs.^32^, ^33^, ^34^, ^35^, ^36^ The choice of model should reflect local resources, workforce expertise, and the preferences and needs of people diagnosed with AYA cancer.

Effective symptom management also depends on effective communication. Qualitative studies suggest that AYAs generally value clinicians who are approachable, attentive, and willing to spend time discussing symptom concerns. Conversely, dismissive responses or normalization of persistent symptoms may discourage future symptom reporting. Some survivors also report hesitating to discuss ongoing symptoms after treatment has ended because they do not wish to appear ungrateful or burden their clinicians.^4^ Beyond symptom assessment, nurses can normalize discussions about persistent symptoms, educate survivors regarding which symptoms warrant evaluation, advocate for appropriate supportive care referrals, and facilitate communication across the survivorship continuum. Patient-reported outcomes (PROs) may be particularly important in AYA survivorship because symptom communication can be influenced by both patient and clinician factors. Some AYAs may hesitate to report symptoms, while clinicians may unintentionally normalize symptoms as expected consequences of treatment. Routine use of validated PRO measures can create opportunities to identify symptoms that may otherwise remain unaddressed and facilitate conversations between survivors and clinicians.

Looking ahead, several priorities should guide the next steps in AYA symptom science. First, supportive care interventions should be designed around the developmental context and stated preferences of AYAs rather than adapted from studies conducted primarily among older adults. Second, greater emphasis should be placed on scalable interventions that can be delivered remotely while maintaining participant engagement through behavioral support, personalization, and thoughtful intervention design. These interventions may best be introduced to AYA cancer survivors in the context of a holistic survivorship care model of which there are many potentially effective variations. Third, once the efficacy of individual symptom management strategies is established for AYA cancer survivors, attention should be devoted to understanding how symptom management interventions can be integrated into routine oncology practice. Finally, future studies should continue incorporating PROs and qualitative methods to ensure that interventions target outcomes that are meaningful to AYAs.

In conclusion, advances in cancer treatment have appropriately focused on improving survival among adolescents and young adults. Oncology nursing should ensure that survivors are also able to return to school, establish careers, build relationships, and pursue the futures they envisioned before cancer. Achieving this goal will require supportive care interventions that are developmentally appropriate, evidence-based, and designed around the priorities of AYAs themselves. By combining rigorous symptom science with patient-centered intervention design, oncology nurses are uniquely positioned to lead this next chapter in AYA survivorship care.

## Declaration of generative AI and AI-assisted technologies in the writing process

During the preparation of this work, the authors used ChatGPT (OpenAI) to refine the manuscript's conceptual framing and to improve the organization, flow, clarity, and style of the writing. After using this tool/service, the authors reviewed and edited the content as needed and take full responsibility for the content of the published article.

## Funding

This work received no external funding.

## Declaration of competing interest

The authors declare no conflict of interest. The corresponding author, Dr. Knoerl, is an editorial board member of *Asia-Pacific Journal of Oncology Nursing*. He was not involved in the editorial review of or the decision to publish this article. The other author declares no competing interests.
